# A structured registration program can be validly used for quality assessment in general practice

**DOI:** 10.1186/1472-6963-9-241

**Published:** 2009-12-21

**Authors:** Andrea S Fokkens, P Auke Wiegersma, Sijmen A Reijneveld

**Affiliations:** 1 Department of Health Sciences, University Medical Center Groningen, University of Groningen, Groningen, The Netherlands

## Abstract

**Background:**

Patient information, medical history, clinical outcomes and demographic information, can be registered in different ways in registration programs. For evaluation of diabetes care, data can easily be extracted from a structured registration program (SRP). The usability of data from this source depends on the agreement of this data with that of the usual data registration in the electronic medical record (EMR).

Aim of the study was to determine the comparability of data from an EMR and from an SRP, to determine whether the use of SRP data for quality assessment is justified in general practice.

**Methods:**

We obtained 196 records of diabetes mellitus patients in a sample of general practices in the Netherlands. We compared the agreement between the two programs in terms of laboratory and non-laboratory parameters. Agreement was determined by defining accordance between the programs in absent and present registrations, accordance between values of registrations, and whether the differences found in values were also a clinically relevant difference.

**Results:**

No differences were found in the occurrence of registration (absent/present) in the SRP and EMR for all the laboratory parameters. Smoking behaviour, weight and eye examination were registered significantly more often in the SRP than in the EMR. In the EMR, blood pressure was registered significantly more often than in the SRP. Data registered in the EMR and in the SRP had a similar clinical meaning for all parameters (laboratory and non-laboratory).

**Conclusions:**

Laboratory parameters showed good agreement and non-laboratory acceptable agreement of the SRP with the EMR. Data from a structured registration program can be used validly for research purposes and quality assessment in general practice.

## Background

In general practice the use of electronic medical records (EMRs) instead of paper patient records has become more and more common [1-5]. In an EMR data about a patient's medical history, clinical outcomes and demographic information is recorded. It can provide information about guidelines, patient condition, clinical outcomes and treatment [6]. These benefits from the EMR could result in more efficient and better patient care and care management [7,8].

Use of EMRs can not only improve care management, but can also be beneficial for research [9-11]. Data collection from an EMR is less time-consuming than from a paper patient record [12,13]. However, data retrieval from the EMR is still complex [10] and in some cases registration of data is inadequate [14]. Because clinical consultation is a complex interaction between caregiver and patient, in EMRs the information is often recorded as a mixture of free-text and coded data [15]. Registration, here understood to be all or part of patient information, is influenced by personal, cultural, technical, health system and financial factors [9]. Besides registration differences, the types of electronic databases available is large and can vary in methodology, size and type of data collected [9,16]. Variability in record systems and information registration makes efficient and valid data retrieval difficult. In general, EMRs have not been designed to facilitate data retrieval [5].

A structured registration program (SRP) is designed for use in addition to the EMR, and has easy data extraction possibilities. In an SRP data can be entered in a structured way, these systems are often disease specific. SRPs use predefined fields for data entry; data (certain selected parameters) can be entered and extracted in one way only. This results in uniformity of data with easy extraction benefits. With such a program, the caregivers have a good overview of patients with a certain disease over a specific period of time. Due to the easy extraction possibilities data can be used for research, benchmarking and care management purposes. For clinical practice, such a structured registration program means the physician has to use an extra program. Also, the structured set-up may conflict with clinical practice. Because of that, user compliance may be lower despite the advantages. However, as long as other solutions for valid and easy data retrieval are not generally available or applicable, SRP can be used.

To be able to confidently use SRP data, data have to agree between the EMR and SRP programs. But evidence on this concordance is incomplete. Thiru et al reviewed the validity of data in EMRs, using data from questionnaires, consultations, survey data or paper-based information as golden standard, but most of these studies only concerned patient diagnoses as outcomes [16]. The available studies of data extraction or data structuring describe solutions, rather than assessing validity of recorded data [17-19]. One study found a high concordance assessing validity between data manually extracted from an EMR and data that were extracted from the EMR using a computerized method [13]. However, these computerized methods for extraction data from the EMR are not commonly available or applicable yet. In actual practice an additional program, SRP, is still used.

There are several reasons why data in the SRP can differ from those in the EMR: the person entering the data can differ and only specific data gathered at a certain point in time from a particular group of patients are to be used. Differences between the recorded data in the two programs are not unlikely. As evidence on agreement between data from EMRs and SRP programs is lacking, this study was designed to assess such an agreement. Aim of the study is to examine the agreement between data from an SRP and from routine EMRs, to determine whether the use of SRP data for research and benchmarking is justified.

## Methods

### Registration systems and practices

The SRP used in this study; "Diabcare", is an integrated information technology system to monitor diabetes care, based on the St Vincent Declaration (diabetes care and research in Europe, 1990). The system consists of a form with parameter fields, which is to be completed once a year for each diabetes patient. All diabetes relevant parameters can be entered in the form: clinical parameters, results of feet and eye examinations, complications, medication, and relevant health education received (e.g. about foot care, healthy eating) [20]. The SRP data, from practices using the system in addition to their EMR, were compared with the data in their EMR.

We had access to SRP data from 24 practices participating in a diabetes structured-care project (DSC) in the North of the Netherlands. The use of the SRP was part of the diabetes structured-care project. We selected ten practices to participate in this study; for practical reasons selection was based on the use of one of the following EMR systems, Promedico and Microhis. Eight practices were willing to participate. There were no differences between these eight and the sixteen other practices in mean number of patients (1715 vs 1974, *p = 0.41) *and number of diabetes patients per general practitioner (50 vs 60, *p = 0.38)*.

### Data collection

To be able to compare fifteen patients per practice, we randomly selected 20 patients per obtained SRP registration. If a patient could not uniquely be linked in the EMR, one of the extra 5 patients could be used. This was the case for twelve patients (10%). For identifying the same patient in the two programs we used a linking key comprised of two initials, gender, and birth date (month/year). To determine whether data from the same registration date were used in both programs, the registration date, HbA1c, total cholesterol and systolic blood pressure were compared.

Records from 119 patients, mean age of 65.5 (sd = 11.9) years and 52.5% female, were collected for comparison (one person was accidentally collected double). In most cases patient data were available for two consecutive years. However, since some practices entered the project later, for these patients only one data set was available. The SRP data obtained consisted of two records for 65% of the patients and of one record for 35% of the patients. This resulted in 196 records that could be compared between databases.

Data from the EMR were collected within the general practice by two medical graduate students using a structured electronic data entry form (SPSS). Data from the SRP and from the EMR were combined into one SPSS file.

The following laboratory and non-laboratory parameters were collected: HbA1c (glycosylated haemoglobin), fasting glucose, cholesterol total, cholesterol HDL, triglyceride, creatinin, microalbuminuria, blood pressure, weight, height, smoking behaviour, and foot-and-eye examination performed.

A passive informed consent procedure was used for this anonymous data collection, meaning that after being informed patients could object to data collection.

### Statistical analysis

Collected parameters from the EMR and SRP were combined and analyzed in SPSS 12.0. In order to compare registration from SRP and EMR, we first assessed differences in the occurrence of registrations (absent or present) using McNemar tests with Bonferroni correction where p < 0.01 was considered a significant difference. Agreement regarding the occurrence of registrations was calculated using Cohen's Kappa (agreement adjusted for chance agreement).

The registrations that were present in both the EMR and SRP were classified as registrations that agreed perfectly and registrations that differed. Whether differences found were also clinically relevant differences was calculated with Cohen's Kappa by determining agreement in relation to target values (normal or aberrant). Cohen's Kappa's between 0.4 and 0.75 were considered moderate to be good agreement, above 0.75 excellent. Cohen's Kappa corrects for chance agreement, but is affected by the distribution of prevalence in the marginal totals [21,22]. Therefore also proportions of concordance were presented in table 1.

**Table 1:** Agreement between SRP and EMR in percentage and Cohen's kappa.

	Parameter absent in both systems (%)	Parameter present in EMR, but absent SRP (%)	Parameter present in SRP, but absent EMR (%)	Kappa^b^, registration (present/absent)	Registered in both systems and agree perfectly (%)	Registered in both systems, but values differ (%)	Kappa^a, b^, target values (normal/aberrant)	Clinical target values
HbA1c	12	9	6	0.54	68	6	0.96	7.0 mmol/l
Range^d^	0-33	0-31	0-13	-0.06-0.84	56-87	0-13	0.77-1.0	
Fasting glucose	5	9	11	0.19	45	30	0.78	7.0 mmol/l
Range	0-13	0-13	3-33	-0.13-0.76	7-67	10-47	0.25-1.0	
Cholesterol total	13	4	8	0.62	67	8	0.89	5.1 mmol/l
Range	0-30	0-13	0-20	0.32-1.0	52-81	0-22	0.69-1.0	
Chol. HDL	14	5	7	0.62	65	8	0.96	1.0 mmol/l
Range	0-30	0-10	0-22	0.32-1.0	43-81	0-27	0.78-1.0	
Triglyceride	14	4	8	0.63	64	10	0.83	2.0 mmol/l
Range	0-30	0-10	0-20	0.33-1.0	48-81	0-26	0.64-1.0	
Creatinine	13	7	10	0.48	61	8	1.0	200 μmol/l
Range	0-33	0-23	0-31	0.36-0.84	47-73	0-17	1.0	
Microalbuminuria	46	14	7	0.58	#	#	#	-
	12-100	3-40	0-19	0.02-1.0	-	-	-	

Blood pressure	7	16*	5*	0.30	58	14	0.79	160/90 mmHg
Range	0-23	0-46	0-22	-0.03-0.76	30-100	0-42	0.2-1.0	
Weight^c^	16	4*	23*	0.39	42	15		
Range	0-40	0-10	0-59	-0.04-0.87	4-93	0-40	0.97 0.86-1.0	25 Kg/m^2^
Height^c ^(n = 119)	14	10	16	0.35	48	13		
Range	0-33	0-40	0-60	0.69-0.76	7-93	0-33		
Smoking behaviour (n = 119)	4	3*	70*	-0.02	22	2	-	-
Range	0-20	0-7	33-100	-0.14-0.35	0-53	0-7	-	
Feet exam	50	12	16	0.41	#	#	-	-
Range	4-93	0-31	0-37	-0.18-0.66	-	-	-	
Eye exam	32	10*	21*	0.36	#	#	-	-
Range	7-60	0-31	0-48	-0.05-0.91	-	-	-	

^a ^N depends on percentage of registered values.

^b ^If at least one variable is a constant, a Kappa could not be calculated for all practices independently.

^c ^To determine Kappa (target values) for weight and height, BMI was computed.

^d ^Range: the minimal and maximal percentage or kappa per outcome.at practice level.

* Significant difference between EMR and SRP based on McNemar test, p < 0.01

# Not comparable due to different units of measurement

- Not applicable

SRP = Structured registration program, EMR = Electronic medical record, HbA1c = Glycosylated haemoglobin

Furthermore, for the obtained registrations we compared the entry date between the SRP and EMR. As an overview of differences between the eight practices, a range was given for all variables. To assess the differences between the eight practices, they were rank-ordered regarding all assessments of agreement (best performing practice, second best, and so on). Next, Spearman correlation coefficients were computed for rank-orders on the various assessments.

## Results

### Occurrence of registrations

No differences were found in the occurrence of registrations between the SRP and EMR for all the laboratory parameters, but regarding fasting glucose kappa for occurrence of registration was poor (table 1, first 4 columns). The proportion of lacking registrations in both registration programs was, regarding the laboratory parameters, the highest for microalbuminuria (46%).

In the non-laboratory parameters significant differences were found in the occurrence of registrations between the EMR and SRP (table 1, first 4 columns). Blood pressure was registered more frequently in the EMR than in SRP (p = 0.001). The following parameters were registered more frequently in SRP than in the EMR: weight, smoking status and eye examination (all p < 0.001). No significant difference was found in the occurrence of registration for height, but kappa for occurrence of registration was poor (table 1, first 4 columns). The proportion of lacking registrations in both registration programs was, for the non laboratory parameters, the highest for feet examination (50%).

### Values of registrations

The proportions of registrations in the EMR and SRP that had different values regarding the laboratory parameters exceeded the 10% only for fasting glucose (i.e. 30%) (table 1, final 4 columns). Regarding the non-laboratory parameters, the proportions of registrations with different values were all beneath the 15%. For all parameters (laboratory and non-laboratory), agreement in both registrations between clinical target values was excellent (k>0.75). This means that data obtained from both programs resulted in findings with the same clinical meaning.

### Further results

Analyses per practice, of the occurrence and of the values of registrations, did not show systematic differences by practice. No practice scored consistently best in all analyses on agreement between the registration programs. Correlation coefficients between rank-orders for various agreements ranged from 0.061 to 0.707, and were in no case statistically significant.

Most laboratory and non-laboratory values were entered in SRP within twelve months after the EMR. However, of the laboratory parameters, 37 of the 936 (3.9%) values were entered more than twelve months after the EMR date (range 380-889 days); this corresponded with ten patients (8.4%). Of the non-laboratory parameters, 21 values were entered after twelve months (range 378-642 days) after the EMR date; twenty patients (16.8%).

## Discussion

This study in routine general practice showed a good agreement between the SRP and EMR data for the laboratory parameters. No differences were found in the occurrence of registration, and data registered in the EMR and in the SRP had a similar clinical meaning for all laboratory parameters. The agreement between the SRP and EMR data was acceptable for the non-laboratory parameters. Differences were found in the occurrence of registrations of smoking behaviour, weight, eye-examination and blood pressure. Data registered in the EMR and in the SRP also had similar clinical meaning for all the non-laboratory parameters.

The clinical significance was comparable for data collected from the EMR or the SRP. This implies that no parameter variation between registrations differed to such a degree that it would have any impact on decisions in clinical treatment. In the laboratory parameters, only fasting glucose had a percentage of values that differed above the 10%. However, the differences in values of fasting glucose were not of a clinical relevance.

In the non-laboratory parameters some differences in the occurrence of the registrations were found. The differences that we found can be explained by differences in ease of entry of data between the two programs. In SRP smoking behaviour can be marked "yes" or "no", while in the EMR there is no such predefined entry possible. Blood pressure was registered more often in the EMR than in the SRP. A reason for this may be the fact that blood pressure is measured often and not only in a diabetes consult.

Registration in SRP was the same or more complete than it was in the EMR for almost all parameters in our sample. Therefore, the extra workload of data entry in a second program did not result in less frequent registration. However, for practical implications, not only agreement between registration programs is important, but also clinical benefits, workload, and satisfaction need to be taken into account before implementing a registration program. The balance between practical usability and data entry and extraction possibilities is important; for a program to be used in clinical practice, it must meet the demands of the users [12,19,23].

The strengths of this study are its embedding in routine practice, the range of parameters for which comparisons have been made, and the different types of comparisons that have been made. By not only taking into account similarity between values, but also agreement in occurrence of registrations (absent/present) and clinical target values, a broad overview of comparability is provided for both laboratory and non-laboratory data. A limitation of our study is that we cannot exclude the possibility of extraction errors [24]. Even though two persons collected data together and they used a structured electronic entry form, extraction errors are still possible. This could inevitably lead to an underestimation of the real agreement.

Furthermore, our findings may be too optimistic when compared to routine practice since GPs participating in research are usually better motivated. Although in this study main motivation of the participated GPs was the diabetes structured care, the SRP was only part of this project. Our findings regarding absent registrations are comparable with other studies, but some parameters (HbA1c, smoking behaviour) were indeed registered more often in our practice sample [25,26].

SRPs for other diseases than diabetes share many characteristics: ease of use, workload, and the variables to be entered, are all very much comparable. Therefore, we feel that the results presented here can be extrapolated to the use of other disease specific programs. This assumption is further substantiated by the fact that the clinical meaning of the various laboratory parameters were the same; parameters that are also of importance to other diseases.

## Conclusions

Data obtained from an SRP in routine primary care are comparable with data in the EMR and can therefore be used for benchmarking and research purposes. Collecting data with an SRP has the considerable advantage of easy and quick access to the data compared with manual extraction from the EMR. More research and system development is needed to optimise and standardise data registration possibilities that serve both routine care and research in general practice. Data from the structured registration program can be used validly for research purposes and quality assessment in general practice.

## Consent

The Medical Ethics Committee was consulted and agreed on study design (procedure was not necessary).

## Conflict of interests

The authors declare that they have no competing interests.

## Authors' contributions

All three authors made a substantial and significant contribution during the preparatory phase of this manuscript. They have all approved the final manuscript, and accept full responsibility for the design and conduct of the study; they had access to the data and approved the submission of this paper.

## Pre-publication history

The pre-publication history for this paper can be accessed here:

http://www.biomedcentral.com/1472-6963/9/241/prepub
